# Plasmodium vivax Infection Alters Mitochondrial Metabolism in Human Monocytes

**DOI:** 10.1128/mBio.01247-21

**Published:** 2021-07-27

**Authors:** Suelen Queiroz Diniz, Andréa Teixeira-Carvalho, Maria Marta Figueiredo, Pedro Augusto Carvalho Costa, Bruno Coelho Rocha, Olindo Assis Martins-Filho, Ricardo Gonçalves, Dhélio Batista Pereira, Mauro Shugiro Tada, Fabiano Oliveira, Ricardo Tostes Gazzinelli, Lis Ribeiro do Valle Antonelli

**Affiliations:** a Instituto René Rachou, Fundação Oswaldo Cruz, Belo Horizonte, Minas Gerais, Brazil; b Instituto de Ciências Biológicas, Departamento de Bioquímica e Imunologia, Universidade Federal de Minas Gerais, Belo Horizonte, Minas Gerais, Brazil; c Instituto de Ciências Biológicas, Departamento de Patologia Geral, Universidade Federal de Minas Gerais, Belo Horizonte, Minas Gerais, Brazil; d Centro de Pesquisas em Medicina Tropical de Rondônia, Porto Velho, Rondônia, Brazil; e National Institutes of Health, NIAID, Laboratory of Malaria and Vector Research, Rockville, Maryland, USA; Washington University School of Medicine

**Keywords:** malaria, *P. vivax*, metabolism, mitochondria, monocytes, mitochondrial metabolism, reactive oxygen species

## Abstract

Monocytes play an important role in the host defense against Plasmodium vivax as the main source of inflammatory cytokines and mitochondrial reactive oxygen species (mROS). Here, we show that monocyte metabolism is altered during human P. vivax malaria, with mitochondria playing a major function in this switch. The process involves a reprograming in which the cells increase glucose uptake and produce ATP via glycolysis instead of oxidative phosphorylation. P. vivax infection results in dysregulated mitochondrial gene expression and in altered membrane potential leading to mROS increase rather than ATP production. When monocytes were incubated with P. vivax-infected reticulocytes, mitochondria colocalized with phagolysosomes containing parasites representing an important source mROS. Importantly, the mitochondrial enzyme superoxide dismutase 2 (SOD2) is simultaneously induced in monocytes from malaria patients. Taken together, the monocyte metabolic reprograming with an increased mROS production may contribute to protective responses against P. vivax while triggering immunomodulatory mechanisms to circumvent tissue damage.

## INTRODUCTION

Malaria remains one of the most prevalent diseases in mankind. According to the World Health Organization (WHO), in 2019, there were approximately 229 million cases of malaria worldwide. Plasmodium vivax is widely distributed, being responsible for almost half of the cases in Asia and most of the cases in the Americas, and represents an impediment to economic development of the regions where it is endemic (https://www.who.int/news-room/fact-sheets/detail/malaria). In contrast to other human malaria parasites, P. vivax cannot be grown in tissue culture, and there is no satisfactory animal model; thus, the scope of research on its host-pathogen interaction is limited.

The substantial host inflammatory immune response to control the parasite burden during malaria is also responsible for many of the symptoms observed during the disease (1–3). Monocytes play an important role in host defense against the infection, representing the main source of inflammatory cytokines during the acute phase of the disease (4). In human peripheral blood, monocytes are a heterogeneous population and can be divided into three major subsets based on CD14 and CD16 expression. These subsets are referred to as classical (CD14^+^CD16^–^), inflammatory or intermediate (CD14^+^CD16^+^), and patrolling (CD14^low^CD16^+^) monocytes (5, 6). Our group previously demonstrated that monocyte subsets are differently activated during P. vivax infection (7). Interestingly, they also differ in their capacity to produce reactive oxygen species (ROS). Inflammatory monocytes produce more total and mitochondrial ROS than their counterparts and are associated with better control of the parasite during clinical infection as well as in *in vitro* killing assays (7).

Considering that immune responses are closely linked to metabolic regulation, and monocytes are key cells on the P. vivax immune response, we investigated monocyte subset metabolic processes during acute P. vivax malaria. Immune cells switch from a relatively quiescent to a highly active metabolic phase during activation (8). For this conversion to take place, the cell must pass from a catabolic to an anabolic state. In the former, macromolecules are degraded and transported through pathways to generate energy to produce ATP and maintain cellular homeostasis. In the latter, cellular metabolism is reorganized with the purpose of balancing ATP production with the generation of intermediary metabolites required for *de novo* synthesis of macromolecules (9). The mitochondrion is a key organelle in this switch, both establishing and sustaining immune cell phenotypes and their function (9, 10). Recent technological advances have provided highly sensitive metabolomic approaches that allow us to define the alterations in metabolism that occur during immune cell activation as well as to elucidate the mechanisms linking specific metabolites to immune cell effector functions (11).

Here, we compare the metabolic properties of monocytes and their subsets from a cohort of P. vivax*-*infected patients versus healthy donors. We demonstrate that during P. vivax malaria, there is a reprogramming in monocyte metabolism evidenced by impairment of oxidative phosphorylation and augmented mitochondrial membrane potential favoring mROS production, which may contribute to parasite control.

## RESULTS

### P. vivax infection alters the metabolic properties of monocytes.

**FIG 1:**
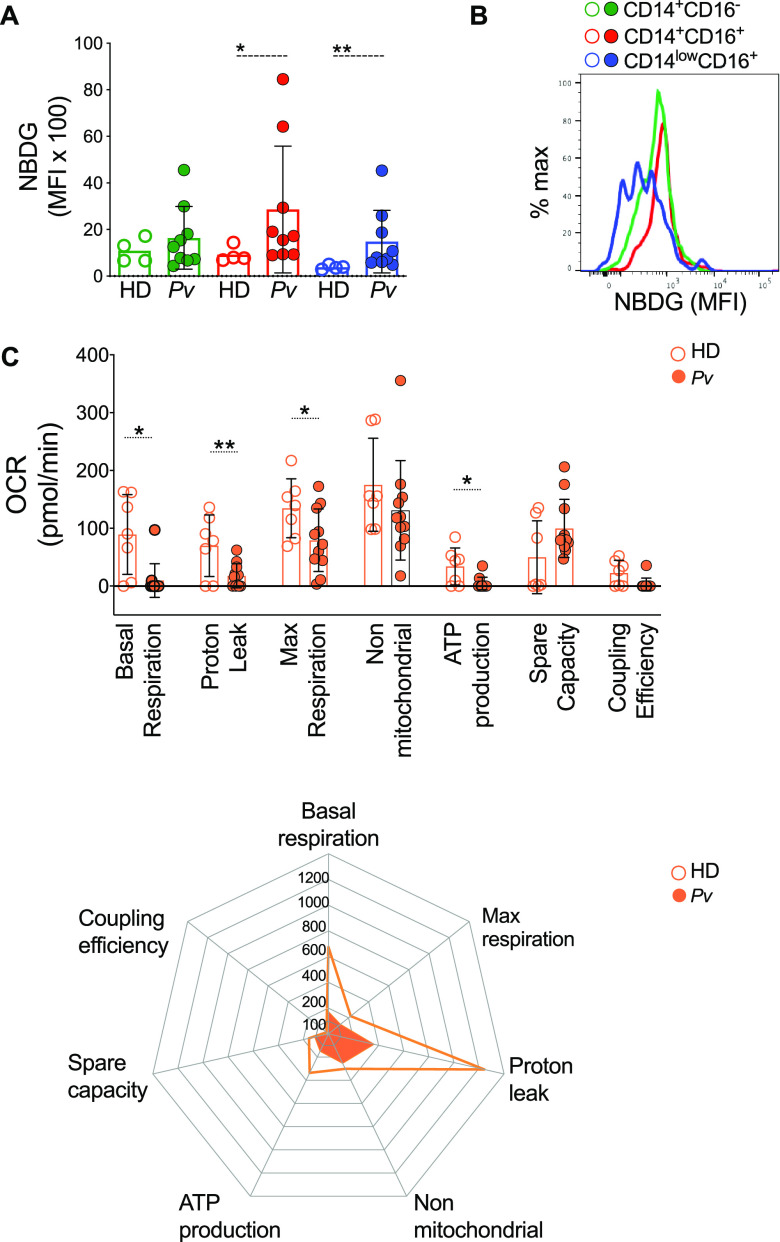
Glucose uptake was increased in monocyte subsets during P. vivax infection. (A) Glucose uptake measured *ex vivo* in CD14^+^CD16^–^ (green), CD14^+^CD16^+^ (red), and CD14^low^CD16^+^ (blue) monocytes from HD (open symbols) and Pv (solid symbols). (B) Level of glucose uptake by monocyte subsets from malaria patients. (C) Extracellular flux analysis using OCR to measure bioenergetics parameters associated with mitochondrial function. Injections of oligomycin, FCCP, and rotenone plus antimycin A were used to perturb mitochondrial functions in circulating monocytes from HD and Pv stimulated with PMA. Radar graph of HD (open orange circles) and Pv (solid orange circles) shows mitochondrial stress parameters normalized to baseline for nonstimulated cells. Cumulative data of at least four individual experiments. Scatterplot with bars representing the mean ± standard deviation (SD) and histogram representing median fluorescence intensity (MFI) of NBDG on the *x* axis; each curve represents a monocyte subset of a single donor normalized to mode 100% on the *y* axis. *P* values were calculated by two-tail Mann-Whitney test (A, HD *n* = 4, Pv *n* = 10; C, HD *n* = 7, Pv *n* = 11). *, *P* ≤ 0.05; **, *P* ≤ 0.01.

Metabolic changes occur upon stress, with the purpose of host adaptation and a shift from oxidative phosphorylation (OXPHOS) to glycolysis following cell activation and have been adopted as a paradigm in the immunology field. However, this phenomenon seems to be both cell and stimulus dependent (12). Considering that monocytes from P. vivax-infected patients (Pv) are favoring the production of mROS, we further expose their metabolic program. Since glucose is an important source of ATP during the activation of immune cells, we evaluated the levels of glycolysis in monocytes from Pv or healthy donors (HD) by comparing glucose uptake (Fig. 1A and B). Inflammatory and patrolling monocytes from Pv displayed increased glucose uptake compared to monocytes from HD (Fig. 1A).

To explore mitochondrial function, we employed a cell mito stress test. Extracellular flux analysis was performed on purified circulating monocytes from HD (open circles) and Pv (solid circles) stimulated with phorbol myristate acetate (PMA). Basal and real-time oxygen consumption rate (OCR) in response to treatment with modulators of respiration [oligomycin, carbonyl cyanide-4-(trifluoromethoxy) phenylhydrazone (FCCP), and rotenone plus antimycin A] were used to assess bioenergetics parameters (Fig. 1C). Significantly higher levels of basal and max respiration, proton leak, and ATP production were observed in HD than in Pv monocytes (Fig. 1C, open circles), indicating that oxygen consumption is being used to produce higher levels of ATP in an efficient manner in HD compared to Pv monocytes.

### P. vivax alters mitochondrial membrane potential in monocyte subsets.

**FIG 2:**
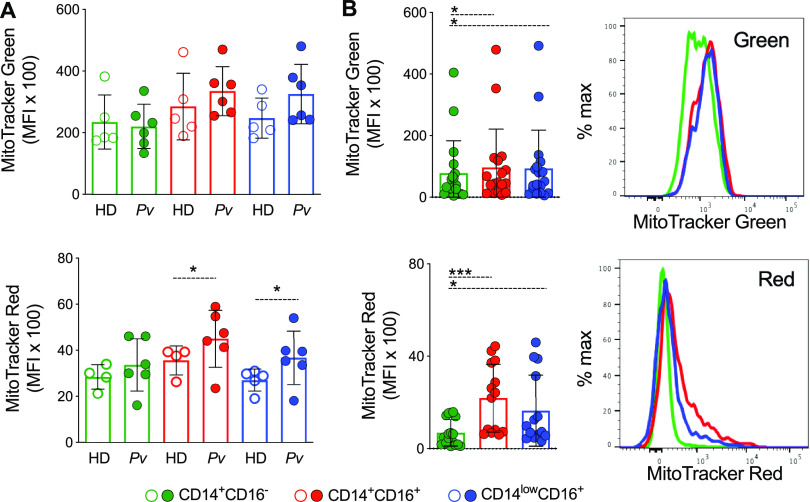
Plasmodium vivax infection alters mitochondrial contents in monocyte subsets. (A) Bar graphs showing *ex vivo* mitochondrial content and mass based on MFI of MitoTracker green (bottom panel) and MitoTracker red CMX-Ros (top panel) reactivity, respectively, in CD14^+^CD16^−^ (green), CD14^+^CD16^+^ (red), and CD14^low^CD16^+^ (blue) monocytes from HD (open symbols) and *Pv* (solid symbols). (B) Bar graphs (left panels) and representative histograms (right panel) showing MFI of MitoTracker green (top panel) and MitoTracker red CMX-Ros (bottom panel) reactivity in monocyte subsets from malaria patients. Cumulative data of at least four individual experiments. Scatterplot with bars representing the mean ± SD and histogram representing the MFI of MitoTracker green and MitoTracker red on the *x* axis; each curve represents a monocyte subset of a single donor normalized to mode 100% on the *y* axis. *P* values were calculated by (A) an unpaired *t* test (HD *n* = 5 and *n* = 4, Pv *n* = 6); (B) one-way ANOVA with Bonferroni’s multiple-comparison test (*n* = 18). *, *P* ≤ 0.05; ***, *P* ≤ 0.001.

Since significant alterations in mitochondrial bioenergetics parameters were found, we evaluated the mitochondrial content of monocyte subsets from Pv and HD using MitoTracker green and MitoTracker red CMXRos. The first reagent is used to stain mitochondria in general, and the second is a probe whose accumulation depends on mitochondrial membrane potential. We observed similar reactivity of MitoTracker green in monocyte subsets from Pv and HD (Fig. 2A, upper panel), suggesting that infection does not significantly alter the mitochondrial mass of the cell. However, Pv inflammatory (CD14^+^CD16^+^) and patrolling (CD14^low^CD16^+^) monocytes showed significantly higher reactivity with MitoTracker red compared with HD monocyte subsets (Fig. 2A, lower panel), suggesting an increase in mitochondrial membrane potential in those cells. Among Pv monocyte subsets, classical (CD14^+^CD16^–^) monocytes displayed the lowest reactivity with both MitoTracker green and MitoTracker red, indicating that these subsets have less mitochondrial content and activity than its other counterparts (Fig. 2B).

### Plasmodium vivax alters the transcription of nuclear genes involved in mitochondrial metabolism.

**FIG 3:**
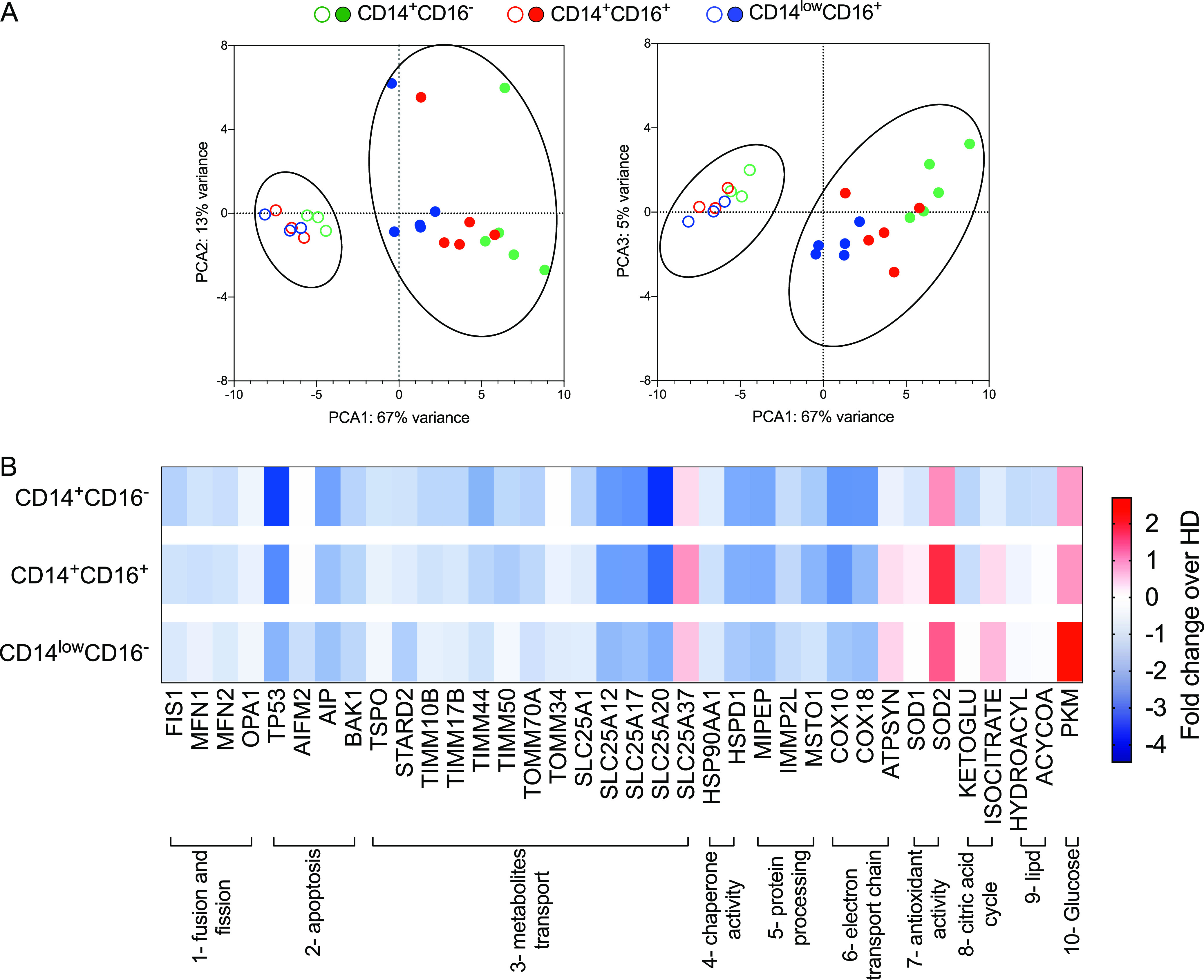
Plasmodium vivax infection disturbs mitochondrial gene expression in monocyte subsets. Gene expression was assessed by nanostring analysis of monocyte subsets from HD and Pv. (A) Principal-component analysis (PCA) for monocyte subsets from HD (open symbols) and Pv (solid symbols). (B) Heatmap representation of 36 differentially regulated genes involved in metabolism clustered in the following 10 groups: (1) fusion and fission, (2) apoptosis, (3) metabolite transport, (4) chaperone activity, (5) protein processing, (6) assembly of the ETC complex IV, (7) antioxidant activity, (8) citric acid cycle, (9) lipid metabolism, and (10) glucose metabolism. Monocytes were harvested in at least 10 different experiments and two sets of samples processed in two independent nanostring runs. PCA coordinates were obtained in limma (A) (HD *n* = 03, Pv *n* = 5). Fold changes between genes differently expressed in Pv and HD (Table S3) grouped from 1 to 7 (except complexes I, II, and III, Cox, and APTsyn) (HD *n* = 3, Pv *n* = 5) and from 8 to 10 and complexes I, II, and III, Cox, and APTsyn (HD *n* = 8, Pv *n* = 8) (B).

Considering that human monocyte subsets differ in their ability to generate mitochondrial ROS when exposed to P. vivax and in order to establish whether this metabolic shift is a consequence of malaria infection, we evaluated the differential expression of genes involved in mitochondrial metabolism from five Pv and three HD using an nCounter nanostring. Selected genes were divided in seven groups based on their function (Table S3). The principal-component analysis (Fig. 3A) clearly shows that Pv and HD monocytes occupy distinct transcriptional spaces on the PC1 and, to a lesser extent, among monocyte subsets on the PC2 and PC3 (Fig. 3A). In the heatmap analysis (Fig. 3B), we depicted the fold changes between genes that displayed significant differences (adjusted *P* < 0.05) in at least one monocyte subset between Pv and HD (Table S3). We observed an overall decreased expression of genes associated with fusion and fission, apoptosis, metabolite transport, chaperone activity, protein processing, and assembly of the electron transport chain (ETC) complex IV, also known as cytochrome *c* oxidase in monocytes from Pv. Contrastingly, SLC25A37, an iron transporter, and superoxide dismutase 2 (SOD2), involved with antioxidant activity, were upregulated in Pv compared to HD. The observed generalized reduction in gene expression in Pv monocytes suggests an altered mitochondrial function in malaria patients. Genes involved in cell metabolism were also analyzed: citric acid cycle (CAC), ETC (additional genes), and lipid and glucose metabolic pathway. A downregulation of genes associated with CAC and glucose metabolism was also observed during malaria. These differences were more frequent among CD14^+^CD16^–^, followed by CD14^+^CD16^+^ monocytes. When we compared monocyte subsets from Pv with those from HD using an ascending gene signature, we confirmed that considerably lower percentages of the monocyte subsets from Pv expressed higher levels of the genes compared to HD (Fig. S1A and B).

10.1128/mBio.01247-21.1 FIG S1 Monocyte subpopulations display distinct mitochondrial gene expression that is further altered upon malaria. The proportion of subjects displaying each gene was assessed using the overall median value, and ascendant signatures were assembled. Those parameters observed in 50% or more of subjects were highlighted in each monocyte subset from healthy donors (A) and P. vivax-infected patients (B). Classical monocytes were represented in green, inflammatory or intermediate monocytes in red, and patrolling monocytes in blue. Genes highlighted in bold represent the parameters observed in 50% or more of subjects in the same monocyte subset from patients and healthy donors. Cumulative data of at least eight individual experiments (HD *n* = 3 to 8, Pv *n*= 5 to 8). Download FIG S1, TIF file, 1.5 MB. Copyright © 2021 Diniz et al. 2021 Diniz et al. https://creativecommons.org/licenses/by/4.0/ This content is distributed under the terms of the Creative Commons Attribution 4.0 International license.

10.1128/mBio.01247-21.7 TABLE S3 Statistical differences between mRNA count in monocyte subsets from healthy donors and P. vivax-infected patients^a^ ^*a*^ Genes were divided into 10 groups: (1, dark pink) fusion and fission, (2, blue) apoptosis, (3, green) metabolite transport, (4, dark orange) chaperone activity, (5, dark green) protein processing, (6, orange) ETC, (7, red) antioxidant activity, (8, purple) Krebs cycle, (9, pink) lipid metabolism, and (10, yellow) glucose metabolism. From 1 to 7 (except complexes I, II, and III, Cox, and APTsyn): HD *n* = 3, Pv *n*= 5; from 8 to 10 and complexes I, II, and III, Cox, and APTsyn: HD *n* = 11, Pv *n*= 13. *P* values were derived from Wald test and adjusted for multiple testing using the procedure of Benjamini and Hochberg. Adj-*P*-values represent differences between HD and Pv considering each monocyte population, and significant differences are depicted in red. Download Table S3, DOCX file, 0.03 MB. Copyright © 2021 Diniz et al. 2021 Diniz et al. https://creativecommons.org/licenses/by/4.0/ This content is distributed under the terms of the Creative Commons Attribution 4.0 International license.

Monocyte subsets from HD share the expression profile of 27 genes (51.92%), compared to only 4 genes (11.11%) in Pv (Fig. S2A). When comparing the monocyte subsets between Pv and HD, we observed that the vast majority of the genes analyzed are exclusively expressed in HD monocytes (Fig. S2B), supporting the hypothesis that P. vivax infection causes substantial changes in monocyte mitochondrial metabolism. Moreover, each monocyte subset from Pv and HD shares distinct gene expression profiles, suggesting that P. vivax infection activates monocyte subsets differentially. Indeed, we observed a decrease in the expression of genes involved on CAC and ETC and an increase in the levels of pyruvate kinase (PKM), the last step of glycolysis, in Pv compared to HD. No changes in genes associated with lipid metabolism were detected.

10.1128/mBio.01247-21.2 FIG S2 Pattern of mitochondrial gene expression in monocyte subsets from healthy donors and P. vivax-infected patients. Venn diagrams were built to identify common gene expression between each group. Only genes expressed in 50% or more of subjects were included. (A) Comparison of gene expression among monocyte subsets from healthy donors (left panel) and Pv-infected patients (right panel). Genes highlighted in bold were commonly expressed by patients and HD when the same pair of monocyte subsets was compared. (B) Comparison of gene expression in each monocyte subset between healthy donors (circle) and malaria vivax patients (square). Genes highlighted in bold were commonly expressed by the three monocyte subsets from patients and/or HD. Genes in italic were exclusively expressed in a specific monocyte subset by Pv and/or HD. Cumulative data of at least eight individual experiments (HD *n* = 3 to 8, Pv *n*= 5 to 8). Download FIG S2, TIF file, 1.2 MB. Copyright © 2021 Diniz et al. 2021 Diniz et al. https://creativecommons.org/licenses/by/4.0/ This content is distributed under the terms of the Creative Commons Attribution 4.0 International license.

### The metabolic shift in monocytes from P. vivax-infected patients is accompanied by induction of SOD2.

**FIG 4:**
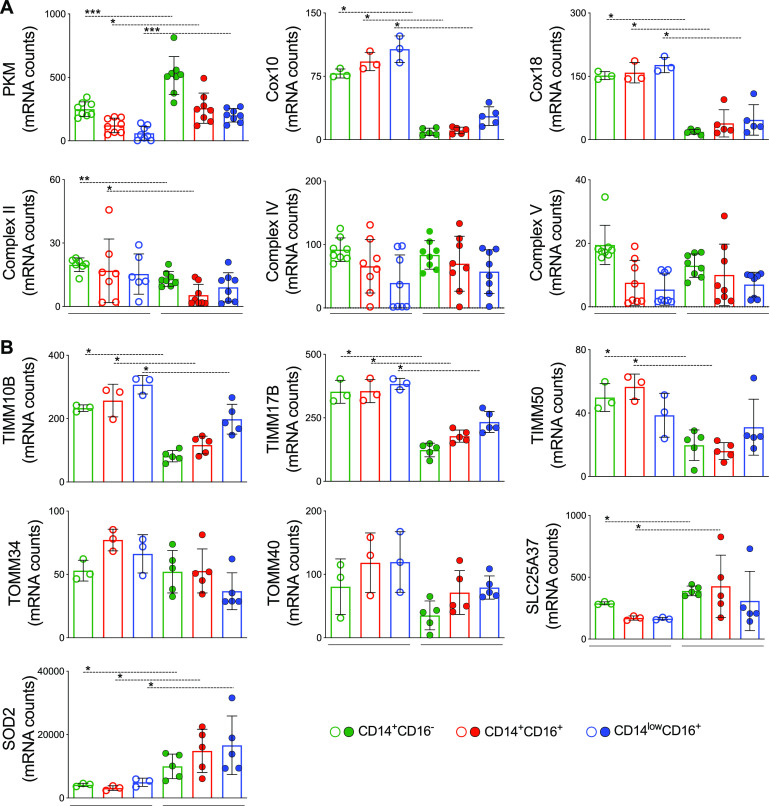
Altered mitochondrial function in monocytes from P. vivax-infected patients is accompanied by induction of PKM, mitoferrin-1, and SOD2. (A) mRNA counts of genes involved in glycolysis (PKM), OXPHOS (complexes II, IV, and V), and complex IV insertion in inner mitochondrial membrane in CD14^+^CD16^–^ (green bar), CD14^+^CD16^+^ (red bar), and CD14^low^CD16^+^ (blue bar) monocytes from Pv and HD. (B) mRNA counts of genes involved in TIM complex and TOM complex assembly, mitoferrin-1 (SLC25A37), and SOD2 in monocyte subsets from Pv and HD. Classical monocytes are represented in green, inflammatory monocytes in red, and patrolling monocytes in blue. Scatterplot with bars representing the mean ± SD. *P* values were calculated by two-tailed Mann-Whitney test. (A) PKM, complexes II, IV, and V (HD *n* = 7 or 8, Pv *n* = 8), COX 10 and 18 (HD *n* = 3, Pv *n* = 5); (B) (HD *n* = 3, Pv *n* = 5). *, *P* ≤ 0.05; **, *P* ≤ 0.01; ***, *P* ≤ 0.001.

We examined in more detail the genes involved in glycolysis and cell respiration in each monocyte subset. As mentioned above, PKM was significantly increased during malaria in all monocyte subsets from Pv compared with HD (Fig. 4A). On the other hand, complex II levels, also known as succinate dehydrogenase (SDH), were decreased in Pv monocytes, while no alteration was observed in complexes IV and V, known as ATP synthase from the ETC (Fig. 4A), compared to HD. Nevertheless, mRNA levels for Cox10 and Cox18, essential for the insertion of complex IV in the mitochondrial inner membrane, were decreased in the three monocyte subsets from Pv versus their counterparts in HD (Fig. 4A).

In addition, we observed a decrease in TIMM10B, TIMM17B, and TIMM50 and an increase in SLC25A37 (Fig. 4B), which are all responsible for metabolite transport between the intermembrane space and the mitochondrial matrix in monocytes from Pv. In contrast, no significant differences were observed in genes belonging to the translocase of the outer membrane (TOM) complex (Fig. 4B) involved in metabolite transport from the cell cytoplasm to the intermembrane space.

Next, we examined the expression of the mitochondrial antioxidant enzyme SOD2. We found that SOD2 is highly expressed in all three monocyte subsets from Pv (Fig. 4B). These data agree with the signature analysis (Fig. S1B) that shows that most Pv (>75%) produce SOD2 levels above the global median of those of the individuals included in the study. Taken together, these results show that the increase in glycolysis in monocytes from *Pv* is accompanied by increased expression of SOD2.

### mROS is produced by mitochondria colocalized with phagolysosomes containing P. vivax-infected reticulocytes (Pv-RET).

**FIG 5:**
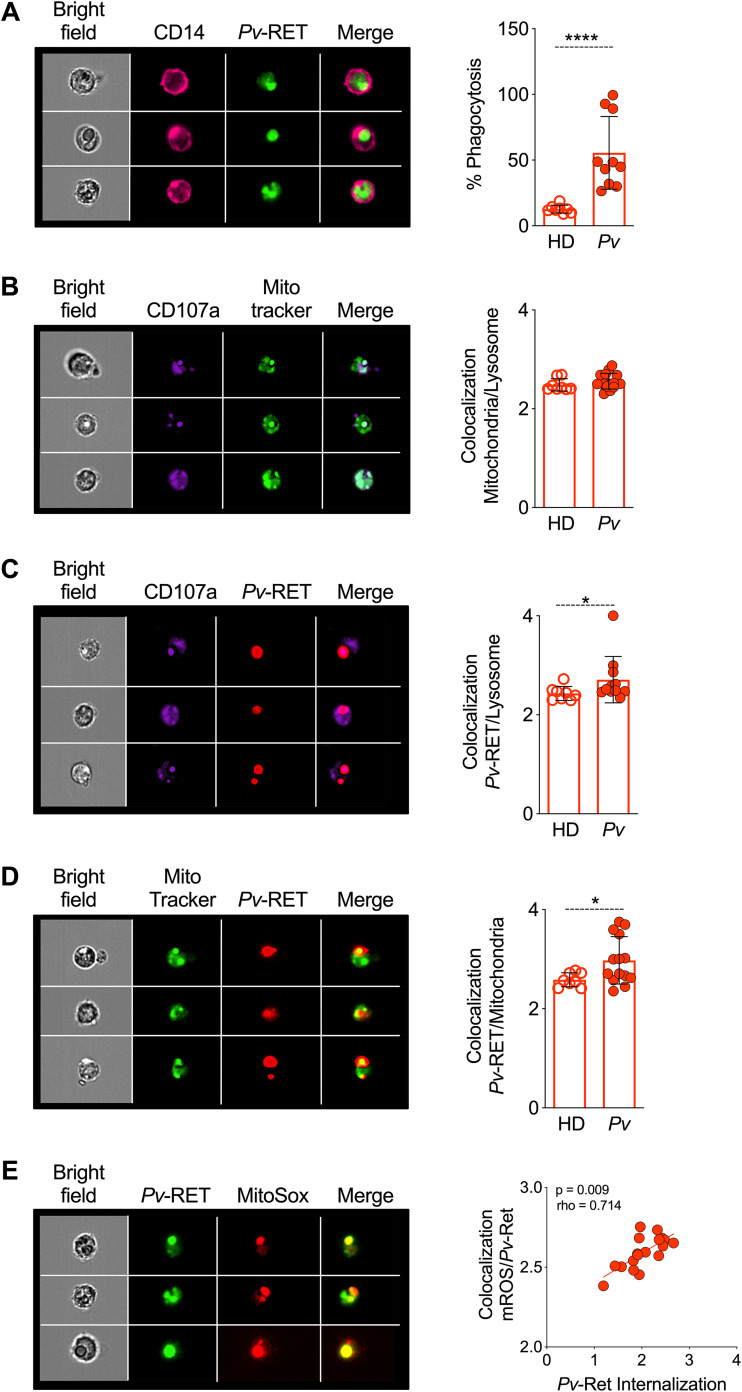
Mitochondria migrate to phagolysosomes containing Pv-RET. Left-hand panels show representative images of Pv, and the right-hand panels show the scatterplot graphs; open and filled symbols represent, respectively, HD and Pv (A to D). (A) Phagocytosis of Pv-RET (green) by monocytes (CD14, pink) from HD and Pv (right panel). (B) Colocalization of phagolysosome (aCD107a, purple) and mitochondria (MitoTracker, green) in monocytes from HD and Pv (right panel). (C) Colocalization of phagolysosome and Pv-RET (red) in monocytes from HD and Pv (right panel). (D) Colocalization of mitochondria (MitoTracker, green) and Pv-RET (red) (left panel) in monocytes from in HD and Pv. (E) Colocalization of Pv-RET (green) and mROS (MitoSox, red) in monocytes from Pv (left panel). Correlation between Pv-RET internalization and colocalization of Pv-RET and MitoSox (right panel). Cumulative data of at least eight individual experiments. Scatterplots with bars representing the mean ± SD. *P* values were calculated by (A to D) two-tailed Mann-Whitney test (A, HD *n* = 8, Pv *n* = 10; B and D, HD *n* = 8, Pv *n* = 15; C, HD *n* = 8, Pv *n* = 11) and (E) Spearman correlation test (HD *n* = 8, Pv *n* = 10). *, *P* ≤ 0.05; ****, *P* ≤ 0.001.

Since the metabolic state of monocytes from Pv are altered compared to those of HD, we examined the impact on phagocytosis of P. vivax-infected reticulocytes (Pv-RET). Image flow cytometry (ImageStream Mark II; Luminex Corporation) was used to assess Pv-RET phagocytosis *in vitro* and colocalization with mitochondria, phagolysosome, and mROS on fluorescence-activated cell sorter (FACS)-purified CD14^+^ monocytes from Pv or HD. Interestingly, monocytes purified from Pv display a greater phagocytosis rate than those from HD (Fig. 5A). mROS has a short half-life, and colocalization of its production site and target increases its functional efficiency (13). Therefore, we assessed the colocalization of mitochondria and phagolysosomes in monocytes. Monocytes from both Pv and HD displayed the same degree of colocalization, indicating that mitochondria are able to migrate to the phagolysosome regardless of the metabolic status of the monocytes (Fig. 5B). When monocytes from Pv were incubated with Pv-RET, they responded with a significantly higher colocalization score on phagolysosomes (Fig. 5C) and mitochondria (Fig. 5D) compared to HD monocytes. We next asked if colocalization of mitochondria with phagolysosome containing Pv-RET would favor mROS production. We assessed colocalization of Pv-RET and MitoSox as well as parasite-phagolysosomal internalization. A strong positive correlation was observed between both parameters, with phagolysosomes containing Pv-RET displaying the highest MitoSox staining (Fig. 5E). Interestingly, inflammatory monocytes from patients displayed higher levels of Pv-RET internalization compared with their counterparts from HD (Fig. S3).

10.1128/mBio.01247-21.3 FIG S3 Inflammatory and patrolling monocytes from patients internalize more Pv-RET than those from HD. (A and B) Pv-RET internalization index (A) by monocyte subsets from Pv patients (Pv, *n* = 06) and (B) by monocyte subsets from P. vivax-infected patients and healthy donors (HD, *n* = 5). (C) mROS production by monocyte subsets from malaria patients. (D) Representative images showing monocyte subsets based on the expression of CD14 (purple), CD16 (pink), and Pv-RET internalization (green). Cumulative data of at least six individual experiments. Scatter plots with bars representing the mean ± SD. *P* values were calculated by (A and C) ANOVA followed by Tukey’s multiple-comparison test (A, Pv *n* = 6; C, Pv *n* = 5) and by Bonferroni’s multiple-comparison test (B) (HD *n* = 5, Pv *n* = 6), and (E) Spearman correlation test (HD *n* = 08, Pv *n* = 10). *, *P* ≤ 0.05; **, *P* ≤ 0.01. Download FIG S3, TIFF file, 2.3 MB. Copyright © 2021 Diniz et al. 2021 Diniz et al. https://creativecommons.org/licenses/by/4.0/ This content is distributed under the terms of the Creative Commons Attribution 4.0 International license.

### Monocytes from P. vivax-infected patients are programmed to produce low levels of ATP.

**FIG 6:**
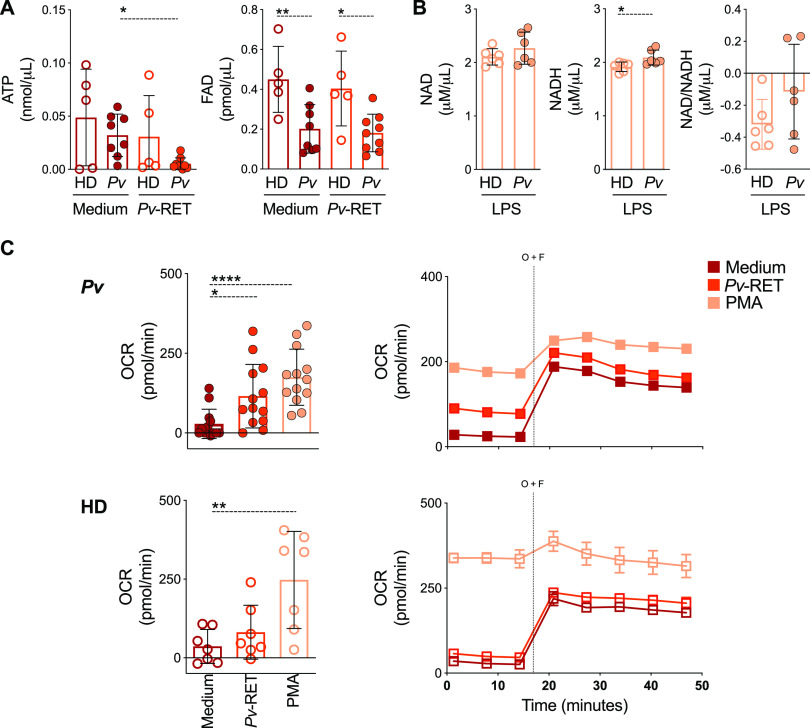
ATP and FAD levels are decreased in P. vivax-infected patients. (A) ATP (left panel) and FAD (right panel) levels were measured in total blood of HD (open symbols) and malaria patients (solid symbols) in the absence (red) and presence (orange) of Pv-RET for 10 min using commercial kits (Abcam). (B) NAD, NADH, and their ratio were measured in PBMC from HD (open symbols) and Pv (solid symbols) and after 10 min of culture in the presence of LPS. (C) Basal oxygen consumption rate (OCR) of monocytes from HD and Pv stimulated with medium (red), Pv-RET (dark orange), or PMA (light orange) (left panel). Basal OCR and real-time changes in OCR assessed during sequential treatment with oligomycin and FCCP (right panel). Cumulative data of at least four individual experiments. The scatterplot with bars representing the mean ± SD and lines shows representative measurements obtained from a single HD and Pv over time. *P* values were calculated by (A and C) one-way ANOVA followed by Bonferroni’s multiple-comparison test (A, HD *n* = 5, Pv *n* = 9 and C, Pv *n* = 13 and HD *n* = 7); (B) two-tailed Mann-Whitney test (HD *n* = 6, Pv *n* = 6). *P ≤ 0.05 **P ≤ 0.01 ***P ≤ 0.001.

Considering that mitochondria are mainly responsible for the generation of metabolic energy in all eukaryotic cells, we evaluated ATP levels in whole-blood samples of Pv in the presence or absence of Pv-RET as a readout of monocyte function. Suramin was added to the samples to block ATP hydrolysis by CD39. The addition of Pv-RET decreased the levels of ATP in Pv cells without significantly altering HD metabolic response (Fig. 6A). Flavin adenosine dinucleotide (FAD) levels were lower in malaria patients even with no further addition of Pv-RET in the culture.

No change was observed in the concentration of NAD (oxidized form) comparing peripheral blood mononuclear cells (PBMC) from Pv and HD (Fig. 6B) when stimulated with lipopolysaccharide (LPS). Although an increase in NADH was observed in cultures of cells from Pv, it did not result in significant differences of the NAD/NADH ratio (Fig. 6B). Together, these results indicate that mitochondria of monocytes from Pv are programmed to produce lower levels of ATP during P. vivax infection compared to those of HD.

We used extracellular flux analysis to further explore the metabolic characteristics of monocytes. Changes in extracellular acidification rate (ECAR) and OCR in response to simultaneous oligomycin and FCCP injections were used to calculate the cellular energy phenotype. Pv and HD monocytes responded similarly to PMA stimulation with an increase in basal OCR (Fig. 6C). Importantly, when cultured with Pv-RET, only monocytes from Pv displayed a statistically significant increase in OCR (Fig. 6C) compared to basal OCR levels, whereas HD monocytes cultured with Pv-RET were no different from basal OCR.

## DISCUSSION

Immunometabolism is a research field that provides insights into how the metabolic alterations affect the immune response (8–11, 14). In the present study, we examined immunometabolic signatures of monocytes from patients with malaria caused by P. vivax infection. We showed that malaria decreases OXPHOS and consequently ATP production, leading to an increase in mitochondrial ROS production in a mitochondrial membrane potential-dependent manner. Furthermore, mROS production appeared to be associated with parasite control, highlighting the role of metabolism in the development of protective immunity.

Monocyte subsets display distinct effector functions and differ in their mitochondrial contents. The influence of P. vivax infection on human monocyte subsets has been previously described and includes the production of ROS (4, 7). Although ROS are known as potent mediators of microbial killing (15, 16), the precise role of oxidative stress during parasitic diseases, including malaria, is not completely understood. While some studies suggest that ROS act in host defense against these pathogens, others associate it with disease pathogenesis (17–21). High oxidative stress is observed even during uncomplicated malaria, and measures of distinct oxidants are used as readouts (22). Changes in the redox status contribute to disease manifestations, including hemolysis, generating free heme that acts as proinflammatory molecule causing undesirable toxicity leading to organ, tissue, and cellular injury (23–25). Thus, the effects of ROS in malaria can be both beneficial and detrimental to the host, and this dichotomy depends on their amount and their site of production. ROS are well known for their microbicidal effects through the oxidative burst mediated by NADPH oxidase in phagocytes (26). However, the absence of NADPH oxidase in mice does not alter parasitemia upon infection with different species of *Plasmodium* (27) and is not required for murine cerebral malaria (28). Interestingly, it was shown that the malaria circumsporozoite protein inhibited the respiratory burst in Kupffer cells (29). Thus, these data would suggest that ROS triggered by NADPH oxidase are not essential for parasite control during experimental malaria. Conversely, we have observed that P. vivax also increases NADPH oxidase activity in monocytes (Fig. S4), suggesting that in humans, as opposed to murine models, this process may be involved in the development of a protective immune response.

10.1128/mBio.01247-21.4 FIG S4 P. vivax increases p47phox expression in inflammation and patrolling. Frequencies of NADPH oxidase components p47 phox and p67 phox expressing monocyte subsets measured by flow cytometry. CD14^+^CD16^–^ (green), CD14^+^CD16^+^ (red), and CD14^low^CD16^+^ (blue) monocytes from HD and Pv. Cumulative data of at least five individual experiments. Scatter plot with bars representing the mean ± SD. *P* values were calculated by unpaired *t* test. *, *P* ≤ 0.05. Download FIG S4, TIF file, 0.2 MB. Copyright © 2021 Diniz et al. 2021 Diniz et al. https://creativecommons.org/licenses/by/4.0/ This content is distributed under the terms of the Creative Commons Attribution 4.0 International license.

Although monocytes from P. vivax-infected patients are highly activated, they are not exhausted or more susceptible to cell death than control monocytes (4, 7). This observation led us to explore the possibility that these cells undergo a metabolic shift in performing their effector functions during malaria. *In vitro* stimulation of macrophages with LPS classically increases glycolysis (30–32) and decreases OXPHOS as hallmarks of activated proinflammatory cells (12, 33). A shift from OXPHOS to glycolysis is also observed in human monocytes stimulated with a specific concentration of LPS, but the same is not observed in monocytes stimulated with Toll-like receptor 2 (TLR2) agonist or bacterial lysates (31, 32), indicating that this reprogramming depends on both the level and type of stimulus. In the case of malaria infection, P. vivax causes an increase in glucose uptake by monocytes, particularly by the CD14^+^CD16^+^ and CD14^low^CD16^+^ subsets. It is expected that cells that produce ATP preferentially via glycolysis and not via OXPHOS have a higher mitochondrial membrane potential, which is important for the generation of mROS (34–38). This happens because the energy generated by electron transport via complexes I, III, and IV across the mitochondrial inner membrane is no longer utilized by ATP synthase to make ATP, but instead stimulates the production of mROS.

In fact, at least eight mitochondrial complexes are associated with superoxide production in mammalian cells (36, 39, 40). Analysis of mitochondrial function, using the spare respiratory capacity measurement, shows that monocytes from Pv display the same ability to respond to an energetic demand as those from HD. During malaria, however, monocytes produce less ATP, a state reinforced by a lower proton coupling efficiency observed in monocytes from Pv. Instead, the higher reactivity of P. vivax-exposed monocytes with MitoTracker red CMXRos suggests that the mROS produced by these cells are generated in a mitochondrial membrane potential-dependent manner.

Considering that human monocyte subsets differ in their ability to generate mitochondrial ROS when exposed to P. vivax and that monocytes are glycolytic during malaria, we analyzed genes involved in mitochondrial function and cell metabolism. Mitochondrial metabolic events are tightly controlled in immune cells, as they can have a major impact on cell functions. Overall, our analysis revealed a major distinction between monocytes from malaria patients and HD and, to some extent, among the monocyte subsets. It has been previously demonstrated that different monocyte subsets naturally display distinct metabolic profiles in accordance with their versatility and dynamic properties (5, 41–44). Interestingly and in agreement with the data discussed above, most of the genes associated with mitochondrial function, fusion and fission, apoptosis, metabolite transport, chaperone activity, protein processing, and assembly of the ETC complex IV, were diminished in monocytes from malaria patients. In general, monocytes from P. vivax-infected patients also had low numbers of mRNAs involved in CAC and ETC. On the other hand, PKM was increased in P. vivax-infected patients. PKM is responsible for ATP production in substrate-level phosphorylation, thus supporting our hypothesis that during malaria, monocytes preferentially produce ATP via glycolysis.

Importantly, studies demonstrated that M1 macrophages exhibit a CAC incomplete in two steps, after citrate and after succinate (45). It was demonstrated that LPS diminished macrophage respiration by attenuating succinate dehydrogenase activity (46, 47). Accordingly, monocytes from P. vivax-infected patients display a decrease in succinate dehydrogenase (SDH) levels, also known as complex II of ETC. Complex II catalyzes the oxidation of succinate to fumarate, during which electrons are transported from FADH2 to ubiquinone that is reduced to ubiquinol, and the electron is transferred to complex s to complex III, ubiquinone/cytochrome *c* oxidoreductase. Indeed, we showed diminished FAD levels, prosthetic group in SDH, in blood from malaria patients compared to HD and decreased mRNA levels of the enzyme SDH in monocyte subsets from malaria patients. SDH is the only enzyme that participates in both CAC and ETC. Together, these results revealed a diminished activity of CAC and ETC in all three monocyte subsets from P. vivax-infected patients.

Moreover, we also observed a decrease in the number of transcripts of genes involved in translocase of the inner membrane (TIM) complex assembly. Mitochondria have their own genetic material that encodes a limited set of proteins mainly involved in CAC and ETC. However, the vast majority of proteins found in mitochondria are derived from genomic DNA, translated into the cell cytosol (48), and then transported to the mitochondrial compartment. Several molecules are found in the mitochondrial matrix that are necessary for the performance of CAC and need to be transported via the TIM complex (49). This complex, responsible for transporting proteins from the intermembrane space to the mitochondrial matrix, is downregulated during malaria. In contrast, there was no difference in genes involved in TOM complex assembly in P. vivax-infected patients compared to HD. This complex is responsible for recognizing precursor proteins in the cytosol and transporting them through the pores of the outer membrane of the mitochondria (49).

Malaria infection did not impact the production of mRNA of complex IV, also known as the cytochrome *c* oxidase complex, that mediates the last step of ETC (50), or of complex V, also known as ATP synthase. These findings suggest that monocytes from P. vivax-infected patients would have the same ability to produce ATP via OXPHOS as the corresponding cells from uninfected HD. However, the decreased expression of genes related to the assembly of cytochrome *c* oxidase complex observed in malaria patients argues against this conclusion. Moreover, our data indicate that transport of electrons in ETC results in an increase in the variation of mitochondrial membrane potential, which produces mROS potential energy instead of being used to produce ATP via OXPHOS.

Along with the increase in mROS production, P. vivax infection also triggered an increase in SOD2 expression in all three monocyte subsets from malaria patients. Superoxide dismutase 2 is an enzyme that catalyzes the dismutation of superoxide into molecular oxygen or hydrogen peroxide, representing an important antioxidant mechanism of the mitochondrion (51). Since oxidative stress can damage host tissue, its production must be tightly controlled (52). Thus, it is very likely that SOD2 is produced as a negative feedback antioxidative mechanism for preventing tissue damage during malaria. Interestingly, the augmentation was observed in SLC25A37 expression, a gene encoding mitoferrin-1, and expressed in high levels in red blood cells (reviewed in reference 53). Mitoferrin-1 is localized on the inner mitochondrial membrane and functions as an essential importer of iron. Thus, mitoferrin-1 plays a role in mitochondrial iron homeostasis, preventing damage caused by iron, which may be an issue during phagocytosis and hemolysis of infected red blood cells during malaria.

Another way to avoid tissue damage triggered by infection is to direct the effector mechanism to the intracellular site of the pathogen. Thus, monocytes from Pv display a greater ability to phagocytose Pv-RET than monocytes from HD. Once inside the monocytes, the colocalization of ROS with their targets seems to be necessary for their microbicidal activity due to the relatively short half-life of ROS (13, 16). Importantly, upon infection of monocytes with Pv-RET, mitochondria colocalize with the phagolysosome containing parasites and produce mROS, suggesting their involvement in parasite killing.

So far, our data support the hypothesis that malaria promotes a metabolic shift favoring mROS production by monocytes. Mitochondrial complexes I and III appear to be the most important sources of these metabolic products (54–56). Thus, high levels NADH trigger production of superoxide by the ETC complex I independent of mitochondrial membrane potential (55). Moreover, no alterations were observed in the NAD/NADH ratio, indicating that this axis is not relevant for mROS production. Alternatively, superoxide generation by complex I can be induced by reverse electron transport stimulated by changes in mitochondrial membrane potential (38). During both processes, ATP production is decreased (35). Here, we show that Pv-RET triggers a decrease in ATP levels in peripheral blood, arguing that mitochondria are not preferentially generating ATP during malaria. Furthermore, analysis of extracellular metabolic flux confirmed that upon P. vivax exposure, monocytes display a glycolytic phenotype distinct from that induced by PMA, which triggers an energetic profile.

Taken together, our findings demonstrated that there is a reprogramming in monocyte metabolism triggered by the microenvironment generated during P. vivax infection. Instead of producing high levels of ATP via OXPHOS, monocytes increase their glycolysis rate in order to provide enough energy to maintain their effector functions. Furthermore, the energy derived from the variation in mitochondrial membrane potential generated by electron transport via ETC is utilized to produce mROS, which can efficiently act on P. vivax due to its close contact with phagolysosomes containing the parasite. Continued studies investigating how metabolites are directly linked to immune cell effector functions in malaria should provide valuable new insights into the nature of both host resistance and pathogenesis in malaria infection.

## MATERIALS AND METHODS

### Patients.

A total of 56 patients with uncomplicated malaria caused by P. vivax were enrolled in this study at Centro de Pesquisa em Medicina Tropical de Rondônia (CEPEM) in Porto Velho, Rondônia, an area of endemicity for malaria in the Amazon, Brazil. The group consisted of nine females (16.1%) and 47 males (83.9%) with an age range from 18 to 72 years (38 ± 13.16) (Table S1). Up to 100 ml of peripheral blood was collected after confirmation of P. vivax infection by thick blood smear film. After blood draw, patients were treated according to the Brazilian Ministry of Health (14). Hematological and biochemical analyzes were performed (Table S2). Fifteen healthy donors (HD) living in Belo Horizonte, Minas Gerais, Brazil, and negative for P. vivax infection were also enrolled.

10.1128/mBio.01247-21.5 TABLE S1 Anamnesis and clinical examination (gender, number of previous malaria episodes, parasite load, and symptoms of P. vivax-infected patients). Download Table S1, DOCX file, 0.01 MB. Copyright © 2021 Diniz et al. 2021 Diniz et al. https://creativecommons.org/licenses/by/4.0/ This content is distributed under the terms of the Creative Commons Attribution 4.0 International license.

10.1128/mBio.01247-21.6 TABLE S2 Hematological records^a^^*a*^Hematrocrit (Hct), hemoglobin (Hb), red blood cell count (RBC), mean corpuscular volume (MCV), mean corpuscular hemoglobin (MCH), mean corpuscular hemoglobin concentration (MCHC), white blood cell count (WBC), monocytes (Mon), platelets (PLT), creatinine (CR), bilirubin (Bili), total cholesterol (TC), high-density lipoprotein (HDL), low-density lipoprotein (LDL), triglycerides (Trig), aspartate aminotransferase (AST), alanine aminotransferase (ALT), alkaline phosphatase (ALP), and gamma-glutamyl transferase (GGT) were accessed at patient enrollment. NA, not analyzed. Download Table S2, DOCX file, 0.04 MB. Copyright © 2021 Diniz et al. 2021 Diniz et al. https://creativecommons.org/licenses/by/4.0/ This content is distributed under the terms of the Creative Commons Attribution 4.0 International license.

### Ethics statement.

These studies were performed under protocols reviewed and approved by the Ethical Committees on Human Experimentation from Centro de Pesquisa em Medicina Tropical de Rondônia (CEP-CEPEM 095/2009) and Instituto René Rachou, Fundação Oswaldo Cruz (CEP-CPqRR 2004), the National Ethical Committee (CONEP 15652) from the Ministry of Health, Brazil. Adults 18 years or older were enrolled in the study and provided written informed consent.

### Monocyte purification.

Peripheral blood mononuclear cells (PBMC) were prepared from heparinized venous blood by Ficoll-Hypaque density gradient centrifugation (GE Healthcare Life Sciences). Monocytes were then purified using two protocols. Total monocytes were purified by positive selection by means of magnetic separation using anti-CD14 microbeads according to the manufacturer’s instructions (MACS; Miltenyi Biotec), or CD14^+^CD16^−^, CD14^+^CD16^+^, and CD14^low^CD16^+^ monocyte subsets were purified with a FACSAria II cell sorter (BD Biosciences), using the following antibodies in different combinations: anti-CD14 (clone 61D3)-APC, anti-CD14 (clone 61D3)-PE (eBioscience), anti-CD16 (clone 3G8)-PerCPCy5.5, anti-CD16 (clone 3G8)-PECy7, and anti-CD66b (clone G10F5)-FITC (BD Bioscience). Anti-CD66b gating was used to exclude contamination by neutrophils. Fresh cells were used in all experiments.

### Reticulocyte purification and labeling.

The red blood cell pellet from the Ficoll-Hypaque density gradient centrifugation was harvested, washed three times, and resuspended in RPMI to a final hematocrit of 10%. Then, 5 ml of this suspension was overlaid on 5 ml of a 45% Percoll (Sigma-Aldrich) solution in a 15-ml tube. After centrifugation, floating mature Plasmodium vivax-infected reticulocytes (Pv-RET) were collected, washed, and labeled with fluorescein isothiocyanate (FITC) or Alexa 647 (200 μg/ml). In brief, 10^6^ Pv-RET were suspended in phosphate-buffered saline (PBS) and incubated for 30 min with FITC or Alexa-647 at room temperature, protected from light and under constant agitation. Cells were washed twice and resuspended with appropriate media at different concentrations.

### Glucose uptake.

Next, 90 μl of blood, collected in polypropylene tubes containing citrate, and 10 μl of 2-(N-[7-Nitrobenz-2-oxa-1,3-diazol-4-yl]amino)-2-deoxyglucose (14.60 μM) (2-NBDG; Invitrogen) was added. This commercial kit uses a fluorescently labeled deoxyglucose analog as a probe for the detection of glucose taken up by cells. The samples were gently homogenized, incubated for 30 min in the dark at 37°C, and placed on ice. BD FACS lysing solution (BD Biosciences) was added, and samples were centrifuged at 200 × *g* at 4°C for 5 min. Following resuspension, the antibodies anti-CD14 (clone 61D3)-APC and anti-CD16 (clone 3G8)-PECy7 were added, and the mixture was incubated for 30 min; the cells were washed and resuspended in 2% fetal calf serum (FCS) in PBS and acquired in the BD FACSCelesta flow cytometer.

### Analysis of monocyte metabolism.

Monocyte metabolism was assessed using a Seahorse XF analyzer (Agilent). Monocytes were resuspended in XF assay medium (Agilent) supplemented with 5.5 mM glucose and 1 mM pyruvate. The cells were then plated at 3 × 10^5^/well in XF-96 plates (Seahorse Bioscience) in the absence or presence of Pv-RET (1:1 ratio) or PMA (100 nM) and cultured for 3 and 1 h, respectively. The oxygen consumption rate (OCR; pmol/min) and the extracellular acidification rate (ECAR; mpH/min) were measured using the Seahorse XFp cell energy phenotype test kit (Agilent), employing real-time injections of oligomycin (1 μM) and carbonyl cyanide-4-(trifluoromethoxy) phenylhydrazone (FCCP; 1 μM). For the measurement of mitochondrial stress, the Seahorse XF Cell Mito stress kit (Agilent) was used according to the manufacturer’s instructions, employing the same concentration of oligomycin and FCCP with the addition of rotenone and antimycin A (each at 1 μM). The resulting data were analyzed by means in the Seahorse Wave Desktop software.

### Mitochondrial content analysis.

Analysis of mitochondrial content was performed using the probes MitoTracker green and MitoTracker red CMXRos (Life Technologies). PBMC were incubated for 15 min with MitoTracker green (20 nM) or MitoTracker red (37.5 nM). The cells were then stained with anti-CD14 (clone 61D3)-APC, anti-CD16 (clone 3G8)-PerCPCy5.5, or PECy7 and anti-CD16 (clone 3G8)-FITC (BD Bioscience) for 30 min at room temperature. After washing, cells were acquired using an LSRFortessa flow cytometer and analyzed using FlowJo v9.3.2 (TreeStar).

### Detection of mROS.

Monocytes were washed in PBS and incubated with Pv-RET in a 1:1 ratio for 3 h at 37°C. In the last 30 min, MitoSox (2.5 μM) was added to the cultures. Cells were washed and stained with the antibodies anti-CD14 (clone 61D3)-APC, anti-CD16 (clone 3G8)-PerCPCy5.5, PECy7, or FITC. Cells were acquired using a LSRFortessa flow cytometer and analyzed using FlowJo v9.3.2 (TreeStar).

### mRNA detection.

The mRNA was assessed by nanostring analysis. nCounter CodeSets were constructed for detecting selected human-specific genes (Table S3). A total of 10^4^ cells of each subset were lysed in RLT buffer (Qiagen) supplemented with β-mercaptoethanol. Lysate was mixed with capture and reporter probes, hybridized to the CodeSet for 16 h, loaded onto the nCounter prep station, and quantified with the nCounter digital analyzer (57). Data were normalized in two ways described previously. The first normalization was for small variations utilizing the internal positive controls that are present in each CodeSet. Then the samples were normalized with seven housekeeping genes that were included in the CodeSet. The data were visualized and exported with nSolver software.

### Colocalization/internalization assays.

After purification by cell sorting, monocytes were plated in Hanks balanced salt solution (HBSS) medium and cultured for 3 h in the absence or in the presence of Pv-RET labeled with Alexa-647 or FITC in a 1:1 ratio and immune human serum (10%). The cells were washed and suspended in either 50 μl of MitoTracker green (10 nM) or MitoSox red (2.5 μM) and incubated for 15 or 30 min. Cells were washed, suspended in 50 μl of PBS with 2% FCS, and analyzed on an image flow cytometer (ImageStreamX Mark II; Luminex Corporation). The antibodies anti-CD107a (clone eBioH4A3)-BV450 (BioLegend) and anti-CD14 (clone 61D3)-PECy7, or -efluor450 (eBioscience) were added at the beginning of culture. The resulting imaging data were analyzed using IDEAS v6.1 software. The internalization and colocalization algorithm tools of the software were used to measure both parameters. The internalization score is defined as the ratio of intensity inside the cell and the intensity of the entire cell. Colocalization of two fluorescences is calculated by the software using the similarity bright detail score, which is derived from the nonmean normalized Pearson’s correlation coefficient calculated for pairs of values taken from distinct channels of fluorescent imagery.

### ATP and FAD levels.

ATP and FAD were measured in whole blood of patients infected with P. vivax and HD using commercial kits (Abcam). Briefly, 200 μl of blood was plated in 5-ml tubes; in the case of ATP, suramin (200 μM) was also added to block the action of the ectonucleotidase CD39 responsible for the hydrolysis of ATP to ADP or AMP. Pv-RET (4 × 10^4^) was added, and the suspension was incubated for 10 min. Supernatant aliquots (100 μl) were collected after centrifugation, and proteins were precipitated in 100 μl of acetonitrile followed by vortexing of the samples for 15 s. After a second centrifugation, supernatant was collected and frozen until measurement of ATP or FAD according to the manufacturer’s instructions for each kit.

### NAD/NADH levels.

The measurement of NAD/NADH was performed using a commercial kit (Abcam). After PBMC purification, 2 × 10^6^ cells were cultured in the presence or absence of LPS for 10 min or 1 h. The cells were centrifuged and resuspended in 100 μl of BD FACS lysing solution (BD Bioscience) following the manufacturer’s instructions for 15 min at room temperature. NAD/NADH levels were measured in cell lysates according to the kit manufacturer’s specifications.

### Statistics.

GraphPad Prism software v8.0 was used for statistical analyses. In selecting the particular statistical test to be employed, the variables of independence, normality, and variance were first determined to define the data as either parametric or nonparametric. Nonparametric data were evaluated using the Mann-Whitney test for unpaired samples. Parametric data were assessed by means of unpaired or paired *t* tests. Analysis of variance (ANOVA) followed by Bonferroni’s or Tukey’s test was used for multiple comparison. Differential expression of genes was calculated by linear modeling with the limma package (58). Genes were considered to be differentially expressed when the *P* value was <0.05, and the adjusted *P* value (or false-discovery rate, FDR) was <0.05 (59). Principal-component analysis (PCA) coordinates were obtained in limma and plotted using GraphPad Prism. Correlation analysis was performed using the Spearman correlation test. Differences were considered statistically significant at a *P* value of <0.05.
