# Supplementary figures and images for: Plasmodium vivax Infection Alters Mitochondrial Metabolism in Human Monocytes
Source: mBio. 2021 Jul 27;12(4):e01247-21. doi: 10.1128/mBio.01247-21 (PMC8406267; doi:10.1128/mBio.01247-21)

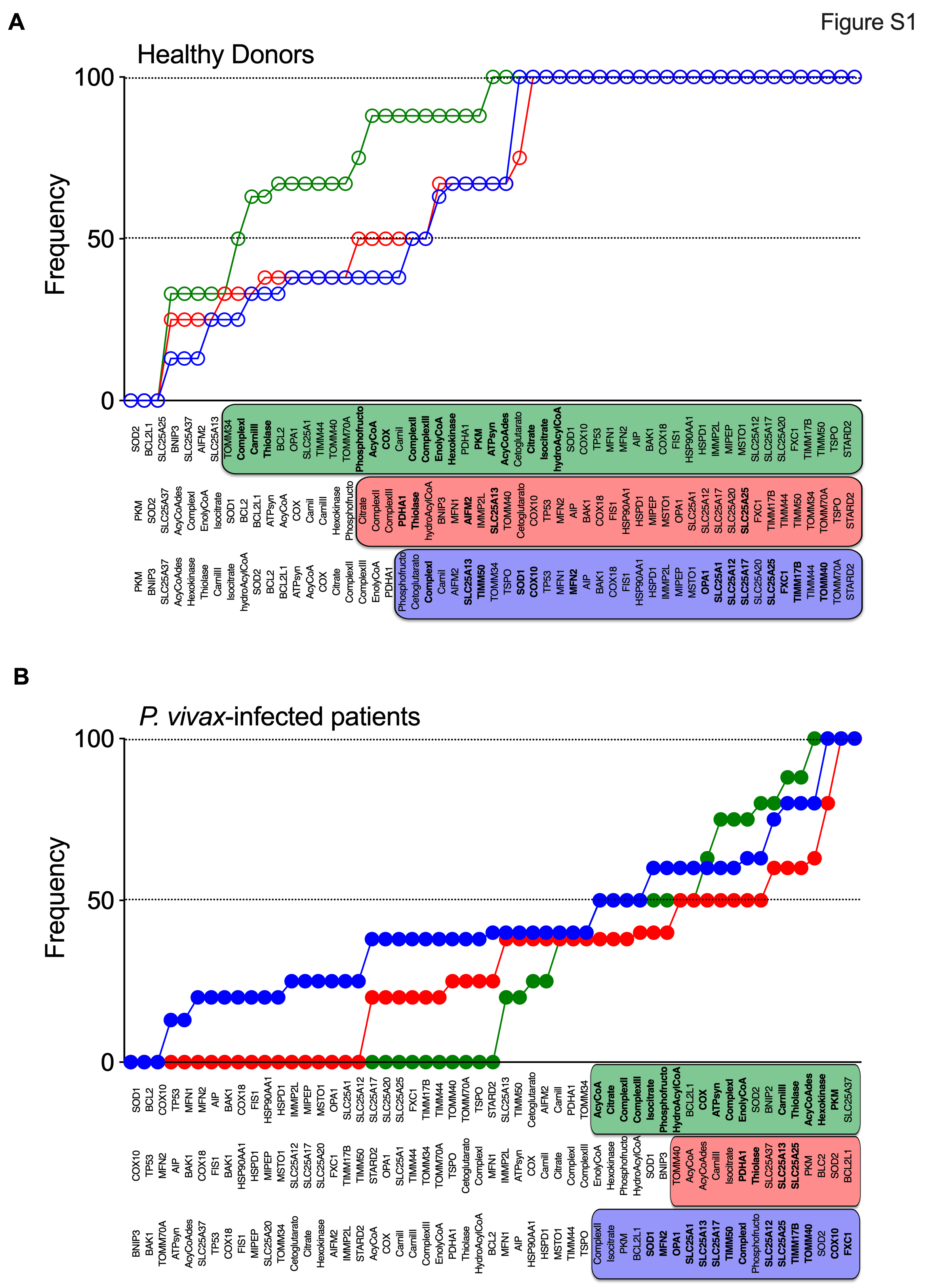

Supplement: FIG S1 [file mbio.01247-21-sf001.tif]

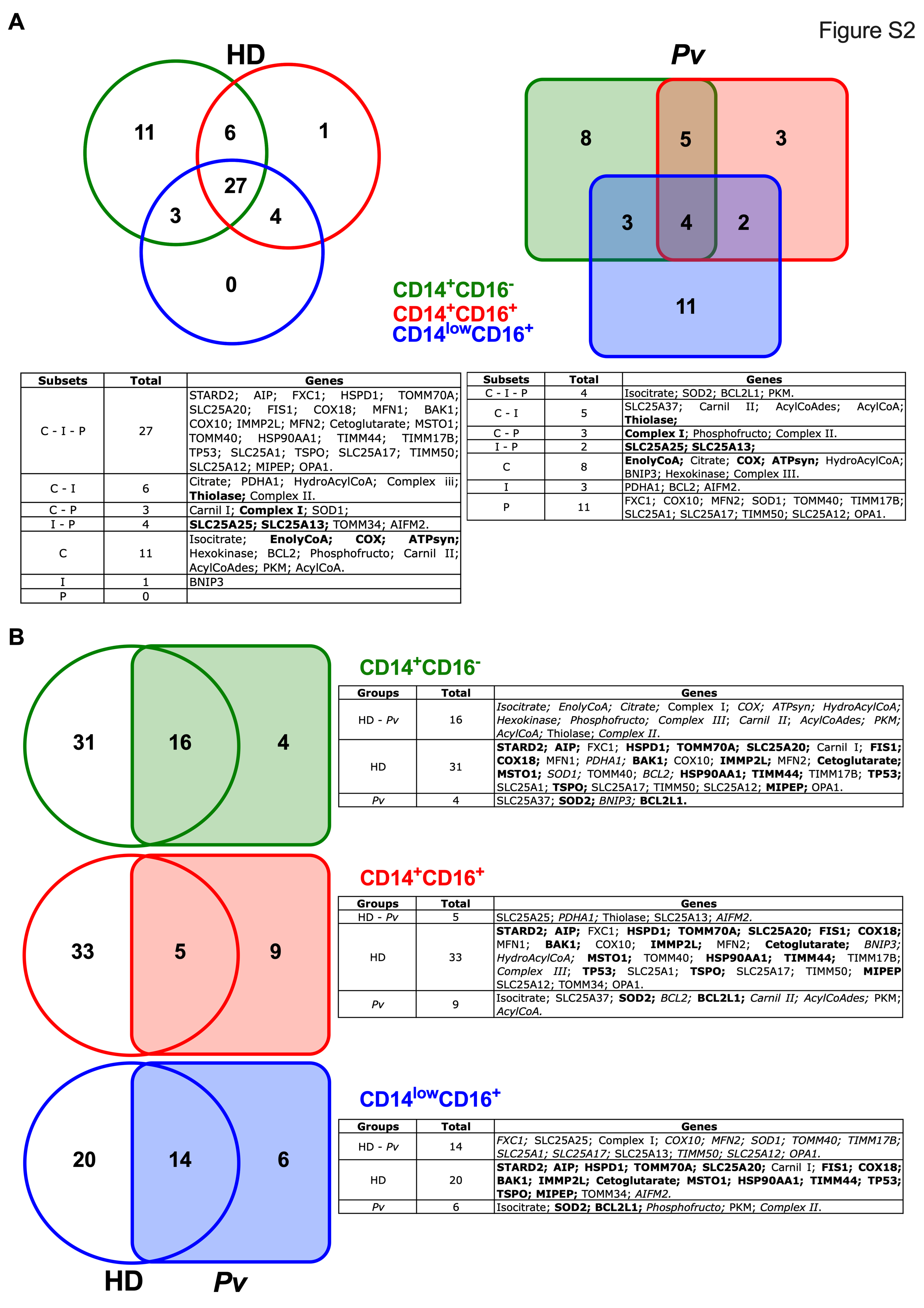

Supplement: FIG S2 [file mbio.01247-21-sf002.tif]

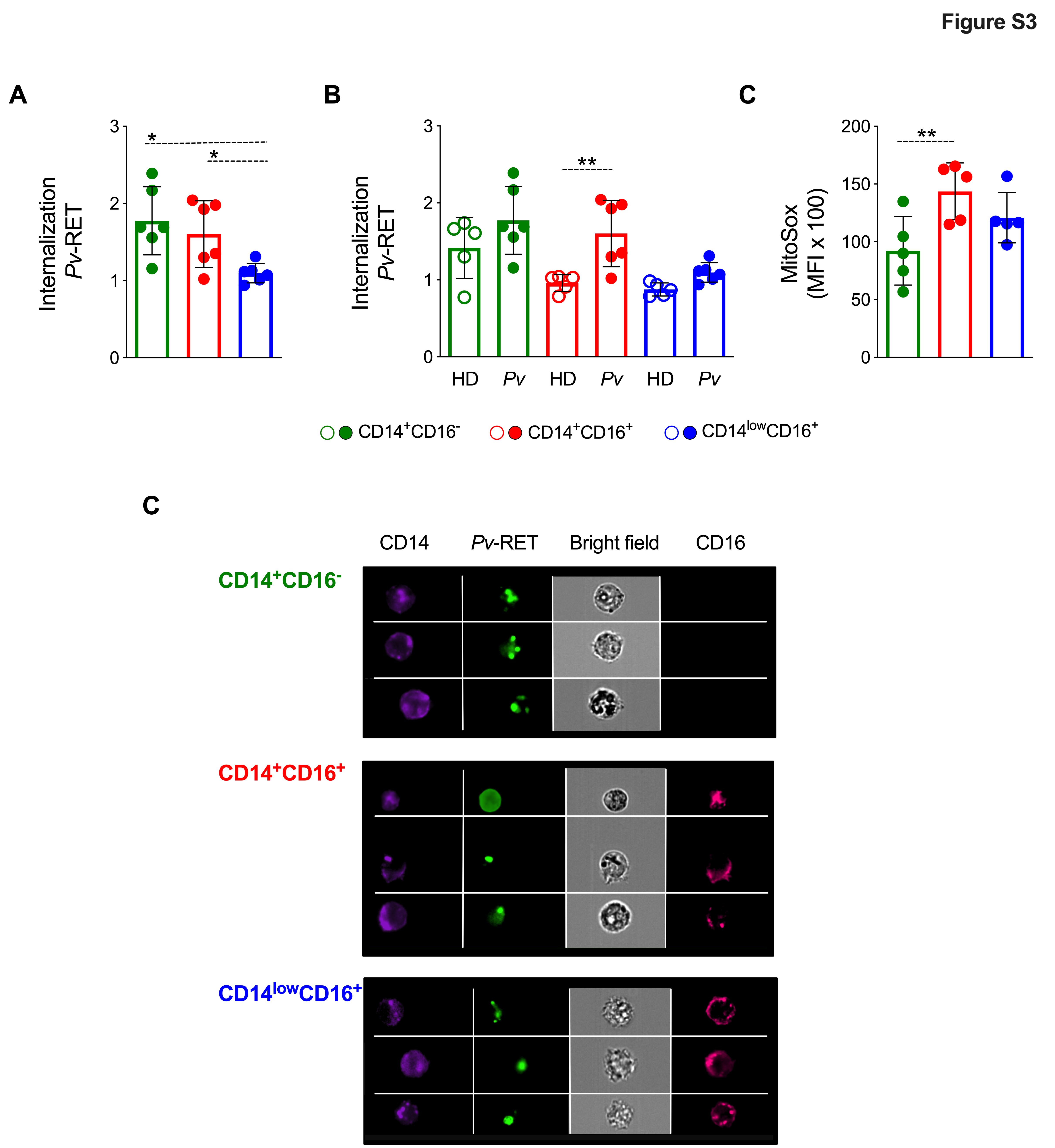

Supplement: FIG S3 [file mbio.01247-21-sf003.tiff]

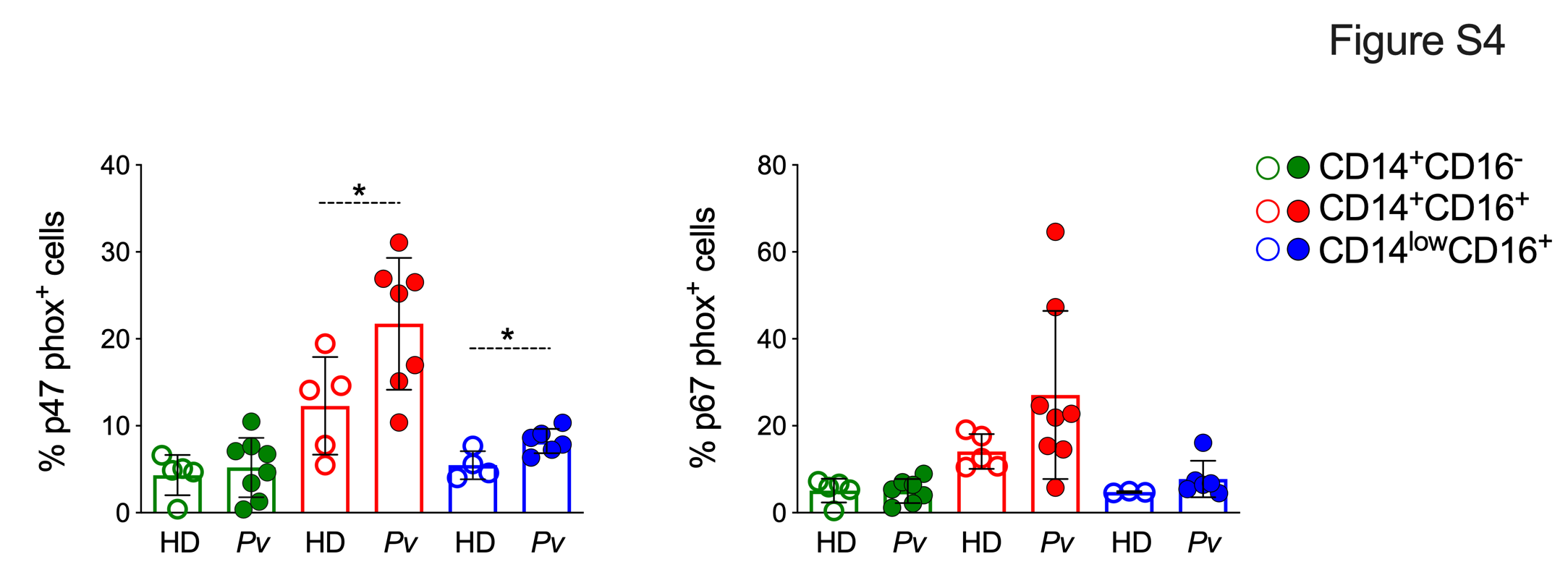

Supplement: FIG S4 [file mbio.01247-21-sf004.tif]
