# Supplementary material for: Plasmodium vivax Infection Alters Mitochondrial Metabolism in Human Monocytes
Source: mBio. 2021 Jul 27;12(4):e01247-21. doi: 10.1128/mBio.01247-21 (PMC8406267; doi:10.1128/mBio.01247-21)
Supplement: TABLE S3 [file mbio.01247-21-st003.docx]

| **Table S3 - Statistical differences between mRNA count in monocyte subsets from healthy donors and *P. vivax*-infected patients** | | | | | |
| --- | --- | --- | --- | --- | --- |
| **Group** | **Official Full Name** | **Gene ID** | **CD14^+^CD16^-^** | **CD14^+^CD16^+^** | **CD14^low^CD16^+^** |
|  |  |  | adj-P-Value | adj-P-Value | adj-P-Value |
| 1 | Fission. mitochondrial 1 | FIS1 | 0.0016 | 0.0025 | 0.0216 |
|  | Mitofusin 1 | MFN1 | 0.0026 | 0.1762 | 0.1380 |
|  | Mitofusin 2 | MFN2 | 0.0012 | 0.0023 | 0.0097 |
|  | Optic atrophy factor 1 | OPA1 | 0.0455 | 0.0447 | 0.1997 |
| 2 | BCL2 apoptosis regulator | BLC2 | 0.0614 | 0.2372 | 0.4045 |
|  | BCL2-like protein 1 | BCL2L1 | 0.7037 | 0.8295 | 0.4574 |
|  | BCL2 interacting protein 3 | BNIP3 | 0.3508 | 0.9600 | 0.0578 |
|  | Tumor protein p56 | TP53 | 0.0000 | 0.0002 | 0.0011 |
|  | Apoptosis inducing factor mitochondria associated 2 | AIFM2 | 0.9532 | 0.7929 | 0.0334 |
|  | Aryl hydrocarbon receptor interacting protein | AIP | 0.0002 | 0.0002 | 0.0014 |
|  | BCL2 antagonist/killer 1 | BAK1 | 0.0003 | 0.0025 | 0.0011 |
| 3 | Translocator protein | TSPO | 0.0032 | 0.0699 | 0.2505 |
|  | STAR related lipid transfer domain containing 2 | STARD2 | 0.0003 | 0.0103 | 0.1041 |
|  | Translocase of inner mitochondrial membrane 10B | TIMM10B | 0.0003 | 0.0036 | 0.0290 |
|  | Translocase of inner mitochondrial membrane 17B | TIMM17B | 0.0004 | 0.0036 | 0.0290 |
|  | Translocase of inner mitochondrial membrane 44 | TIMM44 | 0.0128 | 0.0159 | 0.0290 |
|  | Translocase of inner mitochondrial membrane 50 | TIMM50 | 0.0358 | 0.0010 | 0.4359 |
|  | Translocase of outer mitochondrial membrane 34 | TOMM34 | 0.8316 | 0.1007 | 0.0389 |
|  | Translocase of outer mitochondrial membrane 40 | TOMM40 | 0.1538 | 0.1401 | 0.1792 |
|  | Translocase of outer mitochondrial membrane 70 | TOMM70A | 0.0003 | 0.0092 | 0.0054 |
|  | Solute carrier family 25 member 1 | SLC25A1 | 0.0003 | 0.0159 | 0.0352 |
|  | Solute carrier family 25 member 12 | SLC25A12 | 0.0003 | 0.0002 | 0.0054 |
|  | Solute carrier family 25 member 13 | SLC25A13 | 0.4530 | 0.7929 | 0.8791 |
|  | Solute carrier family 25 member 17 | SLC25A17 | 0.0003 | 0.0002 | 0.0054 |
|  | Solute carrier family 25 member 20 | SLC25A20 | 0.0000 | 0.0005 | 0.0024 |
|  | Solute carrier family 25 member 25 | SLC25A25 | 0.6361 | 0.1250 | 0.4549 |
|  | Solute carrier family 25 member 37 | SLC25A37 | 0.0430 | 0.0480 | 0.2435 |
| 4 | Heat shock protein 90 alpha family class A member 2 | HSP90AA1 | 0.0083 | 0.0024 | 0.0149 |
|  | Heat shock protein family D (Hsp60) member 1 | HSPD1 | 0.0000 | 0.0002 | 0.0011 |
| 5 | Mitochondrial intermediate peptidase | MIPEP | 0.0002 | 0.0019 | 0.0012 |
|  | Inner mitochondrial membrane peptidase subunit 3 | IMMP2L | 0.0015 | 0.0175 | 0.0253 |
|  | Misato mitochondrial distribution and morphology regulator 1 | MSTO1 | 0.0007 | 0.0001 | 0.0225 |
| 6 | NADH:ubiquinone oxidoreductase subunit A13 | Complex I | 0.2005 | 0.9180 | 0.9193 |
|  | Succinate dehydrogenase complex flavoprotein subunit A | Complex II | 0.6802 | 0.9180 | 0.9193 |
|  | Coenzyme Q – cytochrome c reductase | Complex III | 0.2005 | 0.9198 | 0.8479 |
|  | Cytochrome c oxidase | COX | 0.4828 | 0.9198 | 0.7260 |
|  | Cytochrome c oxidase assembly factor COX10 | COX10 | 0.0001 | 0.0001 | 0.0014 |
|  | Cytochrome c oxidase assembly factor COX18 | COX18 | 0.0000 | 0.0029 | 0.0054 |
|  | ATP synthase F0 subunit 6 | ATPsyn | 0.0462 | 0.9180 | 0.8479 |
| 7 | Superoxide dismutase 1 | SOD1 | 0.0014 | 0.7929 | 0.9402 |
|  | Superoxide dismutase 2 | SOD2 | 0.0128 | 0.0024 | 0.0097 |
| 8 | Alpha-ketoglutarate dehydrogenase | ketoglutarate | 0.0010 | 0.2677 | 0.9832 |
|  | Citrate synthase | Citrate | 0.1082 | 0.9180 | 0.9832 |
|  | Aconitase 1 | Isocitrate | 0.0454 | 0.9180 | 0.8479 |
| 9 | Hydroxyacyl CoA dehydrogenase | HydroAcylCoA | 0.0017 | 0.9180 | 0.9193 |
|  | Pyruvate dehydrogenase E1 subunit alpha | PDHA1 | 0.1274 | 0.9180 | 0.9832 |
|  | Thiolase | Thiolase | 0.2005 | 0.9180 | 0.8479 |
|  | Carnitine palmitoyltransferase 1 | Carnil I | 0.1156 | 0.9180 | 0.8634 |
|  | Carnitine palmitoyltransferase 2 | Carnil II | 0.1721 | 0.9180 | 0.9832 |
|  | Enoly-CoA hydratase. short chain 1 | EnolyCoA | 0.6849 | 0.9180 | 0.9832 |
|  | Acyl-CoA dehydrogenase very long chain | AcylCoAdes | 0.2005 | 0.9180 | 0.8479 |
|  | Acyl-CoA synthetase medium chain family member 3 | AcylCoA | 0.0255 | 0.9180 | 0.9832 |
| 10 | Hexokinase 1 | Hexokinase | 0.4482 | 0.9180 | 0.8479 |
|  | Phosphofructokinase | Phosphofructo | 0.2005 | 0.9180 | 0.5053 |
|  | Pyruvate kinase M1/2 | PKM | 0.0010 | 0.2677 | 0.0275 |
