# Supplementary material for: Plasmodium vivax Infection Alters Mitochondrial Metabolism in Human Monocytes
Source: mBio. 2021 Jul 27;12(4):e01247-21. doi: 10.1128/mBio.01247-21 (PMC8406267; doi:10.1128/mBio.01247-21)
Supplement: TABLE S1 [file mbio.01247-21-st001.docx]

| **Table S1**  **Anamnesis and Clinical Examination** | | |
| --- | --- | --- |
| Gender (male, %) | 83.33 |  |
| Age (Mean±SD) | 41.13±12.38 |  |
| Malaria Episodes % | |  |
| 1st | 25.00 |  |
| <5 | 29.17 |  |
| ≥5 | 37.50 |  |
| Parasite/μL | % |  |
| ≤500 | 21.74 |  |
| 500-10000 | 60.42 |  |
| >10000 | 14.58 |  |
| Symptoms | % |  |
| Fever | 80.00 |  |
| Chills | 88.89 |  |
| Nausea | 60.00 |  |
| Vomit | 35.56 |  |
| Diarrhea | 28.89 |  |
| Myalgia | 91.11 |  |
| Headache | 88.89 |  |
| Arthralgia | 82.22 |  |
| Jaundice | 6.52 |  |
