# Supplementary material for: Plasmodium vivax Infection Alters Mitochondrial Metabolism in Human Monocytes
Source: mBio. 2021 Jul 27;12(4):e01247-21. doi: 10.1128/mBio.01247-21 (PMC8406267; doi:10.1128/mBio.01247-21)
Supplement: TABLE S2 [file mbio.01247-21-st002.docx]

**Table S2 – Hemotological Records**

| **Patient ID** | **Hematological Records** | | | | | | | | | | | | | | | | | | | | | | |
| --- | --- | --- | --- | --- | --- | --- | --- | --- | --- | --- | --- | --- | --- | --- | --- | --- | --- | --- | --- | --- | --- | --- | --- |
|  | *Hct^1^ %* | *Hb^2^ g/dL* | *RBC^3^ 10^6^/*  *mm^3^* | *MCV^4^ fL* | *MCH^5^ pg* | *MCHC^6^ %* | *WBC^7^*  *10^3^/*  *mm^3^* | *Mon^8^ %* | *Mon*  */mm^3^* | *PLT^9^*  *10^3^*  */mm^3^* | *CR^10^*  *mg/dL* | *Uric Acid mg/dL* | *Total Bili^11^*  *mg/dL* | *Direct Bili*  *g/dL* | *Indirect Bili*  *g/dL* | *TC^12^ mg/dL* | *HDL^13^ mg/dL* | *LDL^14^ mg/dL* | *Trig^15^ mg/dL* | *AST^16^ U/L* | *ALT^17^ U/L* | *ALP^18^ U/L* | *GGT^19^ U/L* |
| P287 | 41.5 | 14.9 | 4.89 | 84.8 | 30.4 | 35.9 | 9.8 | 7 | 686.7 | 121 | 1.30 | NA | NA | NA | NA | NA | NA | NA | NA | NA | NA | NA | NA |
| P288 | 40.9 | 13.6 | 4.56 | 89.7 | 29.8 | 33.3 | 5.1 | 9 | 461.7 | 181 | 0.99 | NA | 0.78 | 0.23 | 0.55 | 192.7 | 41 | 0 | 437.5 | 21 | 20 | 63 | 74 |
| P292 | 42.1 | 13.7 | 4.97 | 84.7 | 27.6 | 32.5 | 3.5 | 7 | 247.1 | 58 | 0.81 | NA | NA | NA | NA | 135.5 | 3 | 67 | 357.5 | 17 | 29 | 110 | 211 |
| P293 | NA^20^ | NA | NA | NA | NA | NA | NA | NA | NA | NA | 0.60 | NA | 2.61 | 0.59 | 2.02 | 146.9 | 15 | 109 | 112.4 | 25 | 24 | 67 | 47 |
| P294 | 35.9 | 11.8 | 4.30 | 83.4 | 27.4 | 32.8 | 8.5 | 4 | 343.2 | 154 | 0.80 | NA | 1.15 | 0.27 | 0.88 | 162.7 | 32 | 109 | 105.9 | 22 | 18 | 92 | 30 |
| P295 | 41.0 | 12.5 | 4.42 | 92.8 | 29.3 | 30.5 | 4.2 | 10 | 429.0 | 163 | 0.90 | NA | 0.63 | 0.19 | 0.45 | 123.0 | NA | 0 | 488.9 | 19 | 20 | 54 | 40 |
| P297 | 36.7 | 12.1 | 4.63 | 79.3 | 26.1 | 33.0 | 6.8 | 7 | 480.9 | 122 | 0.91 | NA | 2.02 | 1.39 | 0.63 | 63.7 | 3 | 40 | 104.8 | 37 | 40 | 114 | 182 |
| P298 | 47.2 | 15.2 | 5.23 | 90.2 | 29.1 | 32.2 | 3.9 | 2 | 78.4 | 154 | 1.60 | NA | 1.88 | 0.89 | 0.99 | 104.2 | 18 | 65 | 103.5 | 33 | 34 | 109 | 216 |
| P299 | 46.1 | 15.1 | 5.01 | 92.0 | 30.1 | 32.8 | 3.6 | 8 | 289.6 | 90 | 0.78 | NA | 0.59 | 0.33 | 0.26 | 97.5 | 15 | 37 | 284.8 | 43 | 39 | 97 | 81 |
| P300 | 43.3 | 14.5 | 4.74 | 91.4 | 30.6 | 33.5 | 3.8 | 4 | 152.0 | 71 | 0.85 | 3.8 | 1.41 | 0.45 | 0.96 | 132.4 | 38 | 79 | 78.1 | 53 | 44 | 73 | 48 |
| P301 | 45.8 | 14.8 | 5.23 | 87.6 | 28.3 | 32.3 | 4.2 | 14 | 590.8 | 170 | 0.84 | 6.8 | 0.50 | 0.10 | 0.40 | 117.4 | 9 | 80 | 106.5 | 32 | 31 | 69 | 53 |
| P302 | 34.3 | 11.5 | 3.90 | 87.9 | 29.5 | 33.5 | 4.6 | 10 | 467.0 | 138 | 1.06 | 7.2 | 0.90 | 0.33 | 0.57 | 173.2 | 15 | 123 | 176.3 | 18 | 19 | 109 | 120 |
| P303 | 37.9 | 12.4 | 4.64 | 87.3 | 28.6 | 32.7 | 4.1 | 9 | 376.2 | 110 | 0.67 | 3.6 | 1.11 | 0.33 | 0.78 | 100.3 | 13 | 67 | 99.3 | 18 | 18 | 73 | 31 |
| P304 | 53.0 | 17.0 | 6.08 | 87.2 | 28.0 | 32.1 | 7.1 | 10 | 719.0 | 240 | 1.20 | 6.6 | 0.67 | 0.19 | 0.48 | 167.8 | 45 | 103 | 100.7 | 31 | 25 | 84 | 68 |
| P305 | 38.6 | 13.2 | 4.36 | 88.5 | 30.3 | 34.2 | 3.9 | 6 | 234.6 | 89 | 0.82 | 4.6 | 1.57 | 0.55 | 1.02 | 82.1 | 15 | 46 | 167.4 | 25 | 24 | 78 | 25 |
| P306 | 43.6 | 14.6 | 5.26 | 82.9 | 27.8 | 33.5 | 5.0 | 2 | 100.2 | 152 | 1.10 | 5.8 | 1.73 | 1.00 | 0.74 | 107.3 | 10 | 77 | 98.1 | 31 | 23 | 70 | 73 |
| P307 | 36.4 | 12.5 | 4.05 | 90.0 | 31.0 | 34.3 | 8.3 | 9 | 755.1 | 182 | 0.56 | 5.1 | 0.42 | 0.13 | 0.29 | 147.7 | 25 | 106 | 85.4 | 47 | 40 | 84 | 109 |
| P308 | 45.2 | 15.5 | 5.34 | 84.6 | 29.0 | 34.3 | 6.2 | 4 | 251.2 | 171 | 1.08 | 6.6 | 2.93 | 0.33 | 2.27 | 101.8 | 10 | 66 | 131.0 | 23 | 17 | 103 | 48 |
| P309 | 41.8 | 14.7 | 5.03 | 83.1 | 29.2 | 35.2 | 3.0 | 9 | 277.2 | 145 | 1.16 | 5.0 | 0.84 | 0.37 | 0.47 | 115.1 | 9 | 72 | 174.3 | 25 | 29 | 93 | 117 |
| P310 | 40.4 | 14.1 | 4.41 | 91.6 | 32.0 | 34.9 | 7.9 | 7 | 553.7 | 136 | 0.95 | 5.4 | 0.34 | 0.11 | 0.23 | 106.9 | 18 | 72 | 84.7 | 17 | 12 | 55 | 13 |
| P311 | 40.3 | 13.8 | 4.38 | 92.0 | 31.5 | 34.2 | 7.4 | 12 | 895.2 | 401 | 1.01 | 4.8 | 0.46 | 0.16 | 0.30 | 152.1 | 27 | 107 | 93.3 | 18 | 17 | 83 | 13 |
| P312 | 47.7 | 15.4 | 5.55 | 85.4 | 27.7 | 32.5 | 9.9 | 9 | 896.4 | 145 | 1.02 | 5.7 | 2.00 | 0.62 | 1.38 | 111.3 | 3 | 49 | 294.2 | 26 | 21 | 69 | 57 |
| P313 | 40.8 | 13.8 | 4.49 | 90.9 | 30.7 | 33.8 | 9.2 | 7 | 684.2 | 159 | 1.10 | 4.7 | 0.82 | 0.13 | 0.69 | 63.1 | 4 | 38 | 107.7 | 40 | 15 | 72 | 14 |
| P314 | 40.5 | 14.3 | 4.79 | 84.6 | 29.9 | 35.3 | 5.4 | 9 | 491.4 | 152 | 1.38 | 8.6 | 0.80 | 0.37 | 0.44 | 168.8 | 18 | 0 | 606.8 | 95 | 68 | 129 | 410 |
| P315 | 37.1 | 12.6 | 4.72 | 78.6 | 26.7 | 34.0 | 6.0 | 4 | 240.8 | 89 | 0.78 | 4.7 | 0.83 | 0.31 | 0.52 | 73.4 | 15 | 37 | 156.6 | 21 | 16 | 64 | 15 |
| P316 | 43.2 | 14.7 | 4.74 | 91.1 | 31.0 | 34.0 | 4.5 | 8 | 365.6 | 57 | 0.96 | 5.6 | 0.97 | 0.34 | 0.63 | 116.6 | 12 | 52 | 262.2 | 37 | 35 | 60 | 127 |
| P317 | 45.9 | 15.8 | 5.38 | 85.3 | 29.4 | 34.4 | 4.9 | 8 | 393.6 | 118 | 1.01 | 4.5 | 0.92 | 0.29 | 0.64 | 64.8 | 11 | 31 | 113.0 | 21 | 13 | 57 | 11 |
| P318 | 43.6 | 14.9 | 5.04 | 86.5 | 29.6 | 34.2 | 8.8 | 7 | 622.3 | 254 | 0.84 | 4.5 | 1.07 | 0.39 | 0.67 | 67.3 | 9 | 23 | 179.5 | 20 | 11 | 87 | 15 |
| P319 | 48.3 | 15.8 | 5.60 | 86.3 | 28.2 | 32.7 | 8.8 | 9 | 792.9 | 112 | 1.06 | 6.3 | 1.43 | 0.45 | 0.98 | 112.8 | 13 | 50 | 300.5 | 25 | 14 | 69 | 31 |
| P320 | 40.6 | 13.5 | 4.61 | 88.1 | 29.3 | 33.3 | 1.2 | 6 | 77.4 | 104 | 1.12 | 5.9 | NA | NA | NA | 67.4 | 15 | 26 | 169.7 | 23 | 15 | 97 | 18 |
| P321 | 39.1 | 13.4 | 4.43 | 88.3 | 30.2 | 34.3 | 6.5 | 7 | 457.0 | 161 | 0.69 | 4.8 | 0.30 | 0.12 | 0.18 | 131.6 | 15 | 53 | 314.0 | 19 | 17 | 49 | 19 |
| P322 | 43.0 | 14.5 | 4.87 | 88.3 | 29.8 | 33.7 | 10.4 | 7 | 732.9 | 203 | 1.02 | 4.8 | 0.35 | 0.12 | 0.23 | 148.8 | 36 | 91 | 108.2 | 17 | 21 | 84 | 50 |
| P323 | 40.8 | 15.0 | 4.17 | 97.8 | 36.0 | 36.8 | 3.9 | 9 | 358.2 | 73 | 1.22 | 7.1 | 0.65 | 0.15 | 0.50 | 104.9 | 46 | 57 | 157.1 | 27 | 23 | 56 | 17 |
| P325 | 43.6 | 15.5 | 4.86 | 89.7 | 31.9 | 35.6 | 3.6 | 7 | 254.8 | 110 | 1.10 | 6.5 | 1.52 | 0.45 | 1.07 | 105.6 | 17 | 58 | 153.2 | 22 | 30 | 74 | 101 |
| P326 | 48.5 | 16.2 | 5.80 | 83.6 | 27.9 | 33.4 | 6.0 | 7 | 205.8 | 198 | 1.11 | 5.7 | 0.81 | 0.30 | 0.52 | 92.9 | 25 | 46 | 106.4 | 24 | 26 | 55 | 24 |
| P327 | 46.8 | 15.9 | 4.79 | 97.7 | 33.2 | 34.0 | 5.2 | 10 | 528.0 | 249 | 0.81 | 6.3 | 0.73 | 0.22 | 0.51 | 193.5 | 27 | 140 | 133.4 | 30 | 24 | 97 | 28 |
| P328 | 49.0 | 16.7 | 5.60 | 84.5 | 29.8 | 34.1 | 4.4 | 11 | 487.3 | 165 | 0.93 | 2.8 | 0.92 | 0.33 | 0.59 | 134.6 | 44 | 79 | 58.4 | 19 | 21 | 78 | 32 |
| P329 | 33.4 | 10.9 | 3.97 | 84.1 | 27.5 | 32.6 | 2.9 | 26 | 761.8 | 190 | 0.62 | 4.7 | 0.38 | 0.12 | 0.26 | 151.9 | 29 | 88 | 172.6 | 18 | 17 | 85 | 23 |
| P330 | 37.5 | 12.8 | 4.51 | 83.1 | 28.4 | 34.1 | 4.1 | 7 | 288.4 | 126 | 0.73 | NA | 0.46 | 0.18 | 0.29 | 127.3 | 12 | 56 | 297.0 | 31 | 65 | NA | 139 |
| P331 | 44.4 | 14.4 | 5.29 | 83.9 | 27.2 | 32.4 | 5.4 | 8 | 432.8 | 301 | 1.08 | NA | 0.33 | 0.19 | 0.14 | 110.2 | 44 | 33 | 169.9 | 29 | 18 | NA | 84 |
| P332 | 35.5 | 12.5 | 3.76 | 94.4 | 33.2 | 35.2 | 4.3 | 9 | 389.7 | 146 | 0.90 | NA | 1.49 | 0.55 | 0.94 | 120.4 | 18 | 63 | 196.7 | 23 | 15 | 95 | NA |
| P333 | 41.1 | 13.9 | 4.85 | 84.7 | 28.7 | 33.8 | 5.0 | 9 | 457.2 | 181 | 0.84 | NA | 0.37 | 0.13 | 0.24 | 140.5 | 20 | 94 | 130.9 | 22 | 27 | 79 | NA |
| P334 | 48.0 | 16.3 | 5.27 | 91.1 | 30.9 | 34.0 | 8.5 | 4 | 343.2 | 142 | 1.15 | NA | 2.62 | 0.83 | 1.79 | 132.5 | 15 | 49 | 342.7 | 104 | 115 | 100 | NA |
| P339 | 45.5 | 14.3 | 4.70 | 96.6 | 30.4 | 31.4 | 5.3 | 9 | 478.8 | 173 | 0.93 | NA | 0.75 | 0.24 | 0.51 | 180.7 | 32 | 125 | 116.2 | 20 | 28 | NA | NA |
| P340 | NA | NA | NA | NA | NA | NA | NA | NA | NA | NA | 0.71 | NA | 1.70 | 0.50 | 1.20 | 264.3 | 17 | 0 | 856.7 | 57 | 101 | 88 | NA |
| P341 | 39.8 | 12.6 | 4.24 | 93.9 | 29.7 | 31.7 | 3.4 | 18 | 619.2 | 206 | 1.09 | NA | 1.33 | 0.38 | 0.95 | 245.5 | 40 | 137 | 193.0 | 39 | 38 | 100 | NA |
| P342 | 40.2 | 12.8 | 4.33 | 92.8 | 29.6 | 31.8 | 7.8 | 9 | 709.7 | 112 | 0.96 | NA | 0.52 | 0.19 | 0.33 | 87.0 | 14 | 40 | 161.8 | 19 | 20 | 82 | NA |
| P343 | 36.9 | 12 | 3.92 | 94.1 | 30.6 | 32.5 | 4.8 | 9 | 440.1 | 198 | 0.77 | NA | 1.51 | 0.46 | 1.05 | 137.9 | 15 | 90 | 166.4 | 15 | 15 | 93 | NA |

^1^ Hematrocrit (Hct); ^2^ Hemoglobin (Hb); ^3^ Red Blood Cells Count (RBC); ^4^ Mean Corpuscular Volume (MCV); ^5^ Mean Corpuscular Hemoglobin (MCH); ^6^ Mean Corpuscular Hemoglobin Concentration (MCHC); ^7^ White Blood Cells Count (WBC); ^8^ Monocytes (Mon); ^9^ Platelets (PLT); ^10^ Creatinine (CR); ^11^ Bilirubin (Bili); ^12^ Total Cholesterol (TC); ^13^ High Density Lipoprotein (HDL); ^14^ Low Density Lipoprotein (LDL); ^15^ Triglycerides (Trig); ^16^ Aspartate aminotransferase (AST); ^17^ Alanine aminotransferase (ALT); ^18^ Alkaline phosphatase (ALP); ^19^ Gamma-glutamyl transferase (GGT); ^20^ Not analyzed (NA).
